# Resourceful Program Synthesis from Graded Linear Types

**DOI:** 10.1007/978-3-030-68446-4_8

**Published:** 2020-12-29

**Authors:** Jack Hughes, Dominic Orchard

**Affiliations:** King’s College London, London, UK; School of Computing, University of Kent, Canterbury, UK

## Abstract

Linear types provide a way to constrain programs by specifying that some values must be used exactly once. Recent work on *graded modal types* augments and refines this notion, enabling fine-grained, quantitative specification of data use in programs. The information provided by graded modal types appears to be useful for type-directed program synthesis, where these additional constraints can be used to prune the search space of candidate programs. We explore one of the major implementation challenges of a synthesis algorithm in this setting: how does the synthesis algorithm efficiently ensure that resource constraints are satisfied throughout program generation? We provide two solutions to this *resource management* problem, adapting Hodas and Miller’s input-output model of linear context management to a graded modal linear type theory. We evaluate the performance of both approaches via their implementation as a program synthesis tool for the programming language Granule, which provides linear and graded modal typing.

## Introduction

Type-directed program synthesis is a long-studied technique rooted in automated theorem proving [29]. A type-directed synthesis algorithm can be constructed as an inversion of type checking, starting from a type and inductively synthesising well-typed subterms, pruning the search space via typing. Via the Curry-Howard correspondence [21], we can view this as proof search in a corresponding logic, where the goal type is a proposition and the synthesised program is its proof. Recent work has extended type-directed synthesis to refinement types [34], cost specifications [27], differential privacy [35], and example-guided synthesis [12, 33].

Automated proof search techniques have been previously adapted to linear logics, accounting for resource-sensitive reasoning [7–9, 20, 31]. By removing the structural rules of contraction and weakening, linear logic allows propositions to be treated as resources that must be used exactly once [17]. Non-linear propositions are captured via the ‘exponential’ modality !. Linearity introduces a new dimension to proof search and program synthesis: how do we inductively generate terms whilst pruning the search space of those which violate linearity? For example, consider the following inductive *synthesis rule*, mirroring Gentzen’s sequent calculus [15], which synthesises a term of type $A \otimes B$:Reading the rule *bottom up*: from a context of assumptions $\varGamma _{{\mathrm {1}}} , \varGamma _{{\mathrm {2}}}$ we can synthesise the pair $\langle t_{{\mathrm {1}}} , t_{{\mathrm {2}}} \rangle$ from the product type $A \otimes B$ provided that we can inductively synthesise the subterms of the pair, using $\varGamma _{{\mathrm {1}}}$ for the left side and $\varGamma _{{\mathrm {2}}}$ for the right.

But how do we partition a context of free variables $\varGamma$ into $\varGamma _{{\mathrm {1}}}$ and $\varGamma _{{\mathrm {2}}}$ such that $\varGamma _{{\mathrm {1}}}$ contains only those variables needed by $t_{{\mathrm {1}}}$ and $\varGamma _{{\mathrm {2}}}$ only those for $t_{{\mathrm {2}}}$? A naïve approach is to try every possible partition of $\varGamma$. However, this becomes unmanageable as the number of possible partitions is $2^{|\varGamma |}$, i.e., exponential in the number of assumptions. This issue has been explored in automated theorem proving for linear logic, and is termed the *resource management problem* [7].

To address this, Hodas and Miller described an *input-output context management* scheme for linear logic programming [20], further developed by Cervesato et al. [7]. In this approach, synthesis rules take the form $\varGamma \vdash A \Rightarrow t ;\, \varDelta$ with an *input context* $\varGamma$ and an *output context* $\varDelta$ which contains all the hypotheses of $\varGamma$ that were not used in the proof *t* of *A* (akin to the notion of *left over* typing for linear type systems [2, 36]). This output context is then used as the input context to subsequent subgoals. In the case of $A \otimes B$, synthesis has the form:The non-determinism of how to divide $\varGamma$ is resolved by using the entire context as the input for the synthesis of the first subterm $t_{{\mathrm {1}}}$ from type $A$. If this succeeds, the context $\varDelta _{{\mathrm {1}}}$ is returned containing the resources not needed to construct $t_{{\mathrm {1}}}$. These remaining resources provide the input context to synthesise $t_{{\mathrm {2}}}$ from $B$, which in turn returns an output context $\varDelta _{{\mathrm {2}}}$ containing the resources not used by the pair $\langle t_{{\mathrm {1}}} , t_{{\mathrm {2}}} \rangle$. We extend this approach, which we term *subtractive resource management*, to *graded modal types* and present its dual: *additive resource management*. In the additive approach, the output context describes what resources were used to synthesise a term, rather than what may still be used.

Graded modal types comprise an indexed family of modal operators whose indices have structure capturing program properties [32]. In the context of linear logic, graded modalities generalise the indexed modality of Bounded Linear Logic [18] $!_r A$ where $r \in \mathbb {N}$ captures the upper bound *r* on the number of times *A* is used. Generalising such indices to an arbitrary (pre-ordered) semiring yields a type system which can be instantiated to track various properties via the graded modality, a technique which is increasingly popular [4, 13, 14, 16, 24, 25, 32, 36].

Our primary contribution is the extension of the input-output model of resource management for linear program synthesis to graded modal types. Our input and output contexts contain both linear and graded assumptions. Graded assumptions are annotated with a *grade*: an element of a pre-ordered semiring describing the variable’s use. For example, grades drawn from $\mathbb {N}$ yield a system akin to BLL which counts the number of times a variable is used, where a graded assumption $x : [ A ]_{ 2 }$ means *x* can be used twice. An example instantiation of our subtractive pair introduction rule is then as follows: 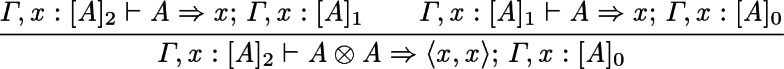


The initial input context contains graded assumption $x : [ A ]_{ 2 }$. The first premise synthesises the term $x$, returning an output context which contains the assumption *x* with grade 1, indicating that $x$ has been used once and can be used one more time. The next premise synthesises the second part of the pair as $x$ using its remaining use. In the final output context, $x$ is graded by 0, preventing it from being used to synthesise subsequent terms.

We adapt the input-output model of linear logic synthesis to subtractive and additive approaches in the presence of graded modal types, pruning the search space via the quantitative constraints of grades. We develop a type-directed synthesis tool for Granule, a functional language which combines indexed, linear, and graded modal types [32]. Granule supports various graded modalities, and its type checker leverages the Z3 SMT solver to discharge constraints on grades [30]. As type-based synthesis follows the structure of types, it is necessary to solve equations on grades during synthesis, for which we make use of Granule’s SMT integration. Such calls to an external prover are costly, and thus efficiency of resource management is a key concern.

Section 2 introduces our core type theory (a subset of Granule’s type system) based on the linear $\lambda$-calculus extended with graded modal types, pairs, and sums. Section 3 describes the two core synthesis calculi (subtractive and additive) as augmented inversions of the typing rules, as well as a variant of additive synthesis. Section 4 describes the implementation^1^ and gives a quantitative comparison of the synthesis techniques on a suite of benchmark programs. The main finding is that the additive approach is often more efficient than the subtractive, presenting a departure from the literature on linear logic theorem proving which is typically subtractive.

Throughout, we will mostly use *types-and-programs* terminology rather than *propositions-and-proofs*. Through the Curry-Howard correspondence, one can switch smoothly to viewing our approach as proof search in logic.

## Graded Linear $\lambda$-calculus

Our focus is a linear $\lambda$-calculus akin to a simply-typed linear functional language with graded modalities, resembling the core languages of Gaboardi et al. [14] and Brunel et al. [4], and a simply-typed subset of Granule [32].

Types comprise linear functions, multiplicative conjunction (product types $\otimes$ and unit 1), additive disjunction (sum types $\oplus$), and a *graded modality* $\Box _r$:where $\square _{ r } A$ is an indexed family of type operators where *r* ranges over the elements of some pre-ordered semiring $({\mathcal {R}}, {*}, {1}, {+}, {0}, {\sqsubseteq })$ parameterising the calculus (where $*$ and $+$ are monotonic with respect to the pre-order $\sqsubseteq$).

The syntax of terms provides the elimination and introduction forms:We use the syntax $()$ for the inhabitant of multiplicative unit $1$. Pattern matching via a $\mathbf{let}$ is used to eliminate products and unit types; for sum types, $\mathbf{case}$ is used to distinguish the constructors. The construct $[ t ]$ introduces a graded modal type $\square _{ r } A$ by ‘promoting’ a term *t* to the graded modality, and $\mathbf {let} \, [ x ] = t_{{\mathrm {1}}} \, \mathbf {in} \, t_{{\mathrm {2}}}$ eliminates a graded modal value $t_{{\mathrm {1}}}$, binding a graded variable *x* in scope of $t_{{\mathrm {2}}}$.

Typing judgments are of the form $\varGamma \vdash t : A$, where $\varGamma$ ranges over contexts:Thus, a context may be empty $\emptyset$, extended with a linear assumption $x : A$ or extended with a graded assumption $x : [ A ]_{ r }$. For linear assumptions, structural rules of weakening and contraction are disallowed. Graded assumptions may be used non-linearly according to the constraints given by their grade, the semiring element *r*. Throughout, comma denotes disjoint context concatenation.

Various operations on contexts are used to capture non-linear data flow via grading. Firstly, *context addition* provides an analogue to contraction, combining contexts that have come from typing multiple subterms in a rule. Context addition, written $\varGamma _{{\mathrm {1}}} + \varGamma _{{\mathrm {2}}}$, is undefined if $\varGamma _{{\mathrm {1}}}$ and $\varGamma _{{\mathrm {2}}}$ overlap in their linear assumptions. Otherwise graded assumptions appearing in both contexts are combined via the semiring $+$ of their grades.

### Definition 1 (Context addition)

For all $\varGamma _{{\mathrm {1}}}, \varGamma _{{\mathrm {2}}}$ *context addition* is defined as follows by ordered cases matching inductively on the structure of $\varGamma _{{\mathrm {2}}}$:

$$\begin{aligned} \varGamma _{{\mathrm {1}}} + \varGamma _{{\mathrm {2}}} = \left\{ \begin{array}{ll} \varGamma _{{\mathrm {1}}} &{} \varGamma _{{\mathrm {2}}} = \emptyset \\ ((\varGamma '_{{\mathrm {1}}}, \varGamma ''_{{\mathrm {1}}}) + \varGamma '_{{\mathrm {2}}}), x : [ A ]_{ ( r + s ) } \; &{} \varGamma _{{\mathrm {2}}} = \varGamma '_{{\mathrm {2}}} , x : [ A ]_{ s } \wedge \varGamma _{{\mathrm {1}}} = \varGamma '_{{\mathrm {1}}} , x : [ A ]_{ r } ,\varGamma ''_{{\mathrm {1}}} \\ (\varGamma _{{\mathrm {1}}} + \varGamma '_{{\mathrm {2}}}), x : A &{} \varGamma _{{\mathrm {2}}} = \varGamma '_{{\mathrm {2}}} , x : A \ \wedge \ x : A \notin \varGamma _{{\mathrm {1}}} \end{array} \right. \end{aligned}$$

In the typing of $\mathbf {case}$ expressions, the *least-upper bound* of the two contexts used to type each branch is used, defined:

### Definition 2 (Partial least-upper bounds of contexts)

For all $$\varGamma _{{\mathrm {1}}}$$, $$\varGamma _{{\mathrm {2}}}$$:

$$\begin{aligned} \varGamma _{{\mathrm {1}}} \sqcup \varGamma _{{\mathrm {2}}} = \left\{ \begin{array}{lll} \emptyset &{} \varGamma _{{\mathrm {1}}} = \emptyset &{} \wedge \; \varGamma _{{\mathrm {2}}} = \emptyset \\ ( \emptyset \sqcup \varGamma '_{{\mathrm {2}}}), x : [ A ]_{ 0 \sqcup s } &{} \varGamma _{{\mathrm {1}}} = \emptyset &{} \wedge \; \varGamma _{{\mathrm {2}}} = \varGamma '_{{\mathrm {2}}} , x : [ A ]_{ s } \\ (\varGamma '_{{\mathrm {1}}} \sqcup ( \varGamma '_{{\mathrm {2}}} , \varGamma ''_{{\mathrm {2}}} )), x : A &{} \varGamma _{{\mathrm {1}}} = \varGamma '_{{\mathrm {1}}} , x : A &{} \wedge \; \varGamma _{{\mathrm {2}}} = \varGamma '_{{\mathrm {2}}} , x : A , \varGamma ''_{{\mathrm {2}}} \\ (\varGamma '_{{\mathrm {1}}} \sqcup ( \varGamma '_{{\mathrm {2}}} , \varGamma ''_{{\mathrm {2}}} )), x : [ A ]_{ r \sqcup s } \;\; &{} \varGamma _{{\mathrm {1}}} = \varGamma '_{{\mathrm {1}}} , x : [ A ]_{ r } &{} \wedge \; \varGamma _{{\mathrm {2}}} = \varGamma '_{{\mathrm {2}}} , x : [ A ]_{ s } , \varGamma ''_{{\mathrm {2}}} \end{array} \right. \end{aligned}$$

where $r\!\sqcup \!s$ is the least-upper bound of grades $r$ and $s$ if it exists, derived from $\sqsubseteq$.

As an example of the partiality of $\sqcup$, if one branch of a **case** uses a linear variable, then the other branch must also use it to maintain linearity overall, otherwise the upper-bound of the two contexts for these branches is not defined.

**Fig. 1.**
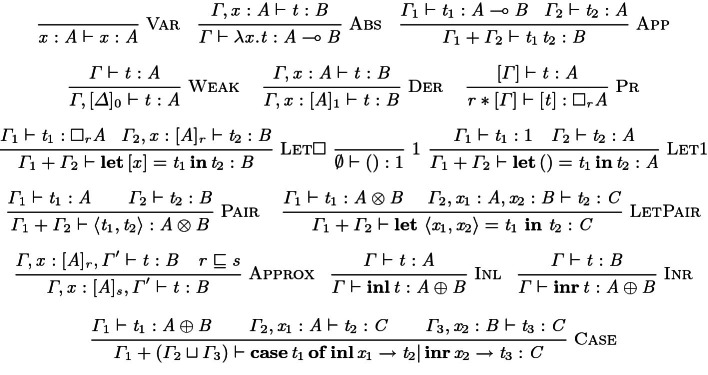
Typing rules of the graded linear $\lambda$-calculus

Figure 1 defines the typing rules. Linear variables are typed in a singleton context (Var). Abstraction (Abs) and application (App) follow the rules of the linear $\lambda$-calculus. Rules for multiplicative products (pairs) and additive coproducts (sums) are routine, where pair introduction ($\textsc {Pair}$) adds the contexts used to type the pair’s constituent subterms. Pair elimination ($\textsc {LetPair}$) binds a pair’s components to two linear variables in the scope of the body $t_{{\mathrm {2}}}$. The $\textsc {Inl}$ and $\textsc {Inr}$ rules handle the typing of constructors for the sum type $A \oplus B$. Elimination of sums ($\textsc {Case}$) takes the least upper bound (defined above) of the contexts used to type the two branches of the case.

The $\textsc {Weak}$ rule captures weakening of assumptions graded by 0 (where $[ \varDelta ]_{0}$ denotes a context containing only graded assumptions graded by 0). Dereliction ($\textsc {Der}$), allows a linear assumption to be converted to a graded assumption with grade 1. Grade approximation is captured by the $\textsc {Approx}$ rule, which allows a grade *r* to be converted to another grade *s*, providing that *r* is *approximated by* *s*, where the relation $\sqsubseteq$ is the pre-order provided with the semiring. Introduction and elimination of the graded modality is provided by the $\textsc {Pr}$ and $\textsc {Let}$ rules respectively. The $\textsc {Pr}$ rule propagates the grade *r* to the assumptions through *scalar multiplication* of $\varGamma$ by *r* where every assumption in $\varGamma$ must already be graded (written $[ \varGamma ]$ in the rule), defined:

### Definition 3 (Scalar context multiplication)

$$\begin{aligned} r * \emptyset = \emptyset \qquad \qquad r * ( \varGamma , x : [ A ]_{ s } ) = ( r * \varGamma ) , x : [ A ]_{ ( r * s ) } \end{aligned}$$

The $\textsc {Let}$ rule eliminates a graded modal value $\square _{ r } A$ into a graded assumption $x : [ A ]_{ r }$ with a matching grade in the scope of the **let** body.

We now give three examples of different graded modalities.

### Example 1

The natural number semiring with discrete ordering $(\mathbb {N}, *, 1, +, 0, \equiv )$ provides a graded modality that counts exactly how many times non-linear values are used. As a simple example, the *S* combinator is typed and defined:

$$\begin{aligned} s&: ( A \multimap ( B \multimap C ) ) \multimap ( A \multimap B ) \multimap ( \square _{ 2 } A \multimap C ) \\ s&= \lambda x . \lambda y . \lambda z' . \; \mathbf {let} \, [ z ] = z' \, \mathbf {in} \, ( x \, z ) \, ( y \, z ) \end{aligned}$$

The graded modal value $z'$ captures the ‘capability’ for a value of type *A* to be used twice. This capability is made available by eliminating $\Box$ (via **let**) to the variable *z*, which is graded $z : [A]_2$ in the scope of the body.

### Example 2

Exact usage analysis is less useful when control-flow is involved, e.g., eliminating sum types where each control-flow branch uses variables differently. The above $$\mathbb {N}$$-semiring can be imbued with a notion of *approximation* via less-than-equal ordering, providing upper bounds. A more expressive semiring is that of natural number intervals [32], given by pairs $$\mathbb {N} \times \mathbb {N}$$ written $$ [ r ... s ] $$ here for the lower-bound $$r \in \mathbb {N}$$ and upper-bound usage $$s \in \mathbb {N}$$ with $$0 = [ 0 ... 0 ] $$ and $$1 = [ 1 ... 1 ] $$, addition and multiplication defined pointwise, and ordering $$ [ r ... s ] \sqsubseteq [ r' ... s' ] = r' \le r \wedge s \le s' $$. Then a coproduct elimination function can be written and typed:

$$\begin{aligned} \oplus _e&: \square _{ [ 0 ... 1 ] } ( A \multimap C ) \multimap \square _{ [ 0 ... 1 ] } ( B \multimap C ) \multimap ( A \oplus B ) \multimap C \\ \oplus _e&= \lambda x' . \lambda y' . \lambda z . \mathbf {let} \, [ x ] = x' \, \mathbf {in} \, \mathbf {let} \, [ y ] = y' \, \mathbf {in} \, ( \mathbf {case} \, z \, \mathbf {of} \, \mathbf {inl} \, u \rightarrow x \, u \mid \, \mathbf {inr} \, v \rightarrow y \, v ) \end{aligned}$$

Linear logic’s exponential !*A* is given by $\square _{ [ 0 ... \infty ] } A$ with intervals over $\mathbb {N} \cup \{\infty \}$ where $\infty$ is absorbing for all operations, except multiplying by 0.

### Example 3

Graded modalities can capture a form of information-flow security, tracking the flow of labelled data through a program [32], with a lattice-based semiring on $\mathcal {R} = \{ \mathsf {Unused} \sqsubseteq \mathsf {Hi} \sqsubseteq \mathsf {Lo} \}$ where $0 = \mathsf {Unused}$, $1 = \mathsf {Hi}$, $+ = \sqcup$ and if $r = \mathsf {Unused}$ or $s = \mathsf {Unused}$ then $r * s = \mathsf {Unused}$ otherwise $r * s = \sqcup$. This allows the following well-typed program, eliminating a pair of $\mathsf {Lo}$ and $\mathsf {Hi}$ security values, picking the left one to pass to a continuation expecting a $\mathsf {Lo}$ input:

$$\begin{aligned} \textit{noLeak}&: ( \square _{ \mathsf {Lo} } A \otimes \square _{ \mathsf {Hi} } A ) \multimap ( \square _{ \mathsf {Lo} } ( A \otimes {1} ) \multimap B ) \multimap B \\ \textit{noLeak}&= \lambda z . \lambda u . \mathbf {let} \ \langle x' , y' \rangle = z \ \mathbf {in} \ \mathbf {let} \, [ x ] = x' \, \mathbf {in} \, \mathbf {let} \, [ y ] = y' \, \mathbf {in} \, u \, [ \langle x , () \rangle ] \end{aligned}$$

*Metatheory.* The admissibility of substitution is a key result that holds for this language [32], which is leveraged in soundness of the synthesis calculi.

### Lemma 1

**(Admissibility of substitution).** Let $\varDelta \vdash t' : A$, then:

- (Linear) If $\varGamma , x : A , \varGamma ' \vdash t : B$ then $\varGamma + \varDelta + \varGamma ' \vdash [ t' / x ] t : B$
- (Graded) If $\varGamma , x : [ A ]_{ r } , \varGamma ' \vdash t : B$ then $\varGamma + ( r * \varDelta ) + \varGamma ' \vdash [ t' / x ] t : B$

## The Synthesis Calculi

We present two synthesis calculi with subtractive and additive resource management schemes, extending an input-output approach to graded modal types. The structure of the synthesis calculi mirrors a cut-free sequent calculus, with *left* and *right* rules for each type constructor. Right rules synthesise an introduction form for the goal type. Left rules eliminate (deconstruct) assumptions so that they may be used inductively to synthesise subterms.

### Subtractive Resource Management

Our subtractive approach follows the philosophy of earlier work on linear logic proof search [7, 20], structuring synthesis rules around an input context of the available resources and an output context of the remaining resources that can be used to synthesise subsequent subterms. Synthesis rules are read bottom-up, with judgments $\varGamma \vdash A \Rightarrow ^{\!-} t ;\, \varDelta$ meaning from the *goal type* $A$ we can synthesise a term $t$ using assumptions in $\varGamma$, with output context $\varDelta$. We describe the rules in turn to aid understanding. The appendix [22] collects the rules for reference.

Variable terms can be synthesised from linear or graded assumptions by rules:On the left, a variable $$ x $$ may be synthesised for the goal $$ A $$ if a linear assumption $$ x : A $$ is present in the input context. The input context without $$ x $$ is then returned as the output context, since *x* has been used. On the right, we can synthesise a variable *x* for *A* we have a graded assumption of *x* matching the type. However, the grading $$ r $$ must permit $$ x $$ to be used once here. Therefore, the premise states that there exists some grade *s* such that grade *r* approximates $$s + 1$$. The grade *s* represents the use of *x* in the rest of the synthesised term, and thus $$ x : [ A ]_{ s } $$ is in the output context. For the natural numbers semiring, this constraint is satisfied by $$s = r - 1$$ whenever $$r \ne 0$$, e.g., if $$r = 3$$ then $$s = 2$$. For intervals, the role of approximation is more apparent: if $$r = [ 0 ... 3 ] $$ then this rule is satisfied by $$s = [ 0 ... 2 ] $$ where $$s + 1 = [ 0 ... 2 ] + [ 1 ... 1 ] = [ 1 ... 3 ] \sqsubseteq [ 0 ... 3 ] $$. Thus, this premise constraint avoids the need for an additive inverse. In the implementation, the constraint is discharged via an SMT solver, where an unsatisfiable result terminates this branch of synthesis.

In typing, $\lambda$-abstraction binds linear variables to introduce linear functions. Synthesis from a linear function type therefore mirrors typing:Thus, $\lambda x . t$ can be synthesised given that *t* can be synthesised from *B* in the context of $\varGamma$ extended with a fresh linear assumption $x : A$. To ensure that $x$ is used linearly by $t$ we must therefore check that it is not present in $\varDelta$.

The left-rule for linear function types then synthesises applications (as in [20]):The rule synthesises a term for type $C$ in a context that contains an assumption $x_{{\mathrm {1}}} : A \multimap B$. The first premise synthesises a term $t_{{\mathrm {1}}}$ for $C$ under the context extended with a fresh linear assumption $x_{{\mathrm {2}}} : B$, i.e., assuming the result of $x_{{\mathrm {1}}}$. This produces an output context $\varDelta _{{\mathrm {1}}}$ that must not contain $x_{{\mathrm {2}}}$, i.e., $x_{{\mathrm {2}}}$ is used by $t_{{\mathrm {1}}}$. The remaining assumptions $\varDelta _{{\mathrm {1}}}$ provide the input context to synthesise $t_{{\mathrm {2}}}$ of type $A$: the argument to the function $x_{{\mathrm {1}}}$. In the conclusion, the application $x_{{\mathrm {1}}} \, t_{{\mathrm {2}}}$ is substituted for $x_{{\mathrm {2}}}$ inside $t_{{\mathrm {1}}}$, and $\varDelta _{{\mathrm {2}}}$ is the output context.

Note that this rule synthesises the application of a function given by a linear assumption. What if we have a graded assumption of function type? Rather than duplicating every left rule for both linear and graded assumptions, we mirror the dereliction typing rule (converting a linear assumption to graded) as:Dereliction captures the ability to reuse a graded assumption being considered in a left rule. A fresh linear assumption $y$ is generated that represents the graded assumption’s use in a left rule, and must be used linearly in the subsequent synthesis of $t$. The output context of this premise then contains $x$ graded by $s'$, which reflects how $x$ was used in the synthesis of $t$, i.e. if $x$ was not used then $s' = s$. The premise $\exists s .\, r \sqsupseteq s + 1$ constrains the number of times dereliction can be applied so that it does not exceed *x*’s original grade *r*.

For a graded modal goal type $\square _{ r } A$, we synthesise a promotion $[ t ]$ if we can synthesise the ‘unpromoted’ $t$ from $A$:Recall that typing of a promotion $[ t ]$ scales all the graded assumptions used to type $t$ by *r*. Therefore, to compute the output context we must “subtract” *r*-times the use of the variables in $t$. However, in the subtractive model $\varDelta$ tells us what is left, rather than what is used. Thus we first compute the *context subtraction* of $\varGamma$ and $\varDelta$ yielding the variables usage information about $t$:

#### Definition 4 (Context subtraction)

For all $\varGamma _{{\mathrm {1}}}, \varGamma _{{\mathrm {2}}}$ where $\varGamma _{{\mathrm {2}}} \subseteq \varGamma _{{\mathrm {1}}}$:


As in graded variable synthesis, context subtraction existentially quantifies a variable *q* to express the relationship between grades on the right being “subtracted” from those on the left. The last conjunct states *q* is the greatest element (wrt. to the pre-order) satisfying this constraint, i.e., for all other $$q' \in \mathcal {R}$$ satisfying the subtraction constraint then $$ q \sqsupseteq q' $$ e.g., if $$r = [ 2 ... 3 ] $$ and $$s = [ 0 ... 1 ] $$ then $$q = [ 2 ... 2 ] $$ instead of, say, $$ [ 0 ... 1 ] $$. This *maximality* condition is important for soundness (that synthesised programs are well-typed).

Thus for R$\square ^{-}$, $\varGamma - \varDelta$ is multiplied by the goal type grade *r* to obtain how these variables are used in $t$ after promotion. This is then subtracted from the original input context $\varGamma$ giving an output context containing the left-over variables and grades. Context multiplication requires that $\varGamma - \varDelta$ contains only graded variables, preventing the incorrect use of linear variables from $\varGamma$ in $t$.

Synthesis of graded modality elimination, is handled by the $\textsc {L}\square ^{-}$ left rule:Given an input context comprising $\varGamma$ and a linear assumption $x_{{\mathrm {1}}}$ of graded modal type, we can synthesise an unboxing of $x_{{\mathrm {1}}}$ if we can synthesise a term $t$ under $\varGamma$ extended with a graded assumption $x_{{\mathrm {2}}} : [ A ]_{ r }$. This returns an output context that must contain $x_{{\mathrm {2}}}$ graded by *s* with the constraint that *s* must approximate 0. This enforces that $x_2$ has been used as much as required by the grade *r*.

The right and left rules for products, units, and sums, are then fairly straightforward following the subtractive resource model:

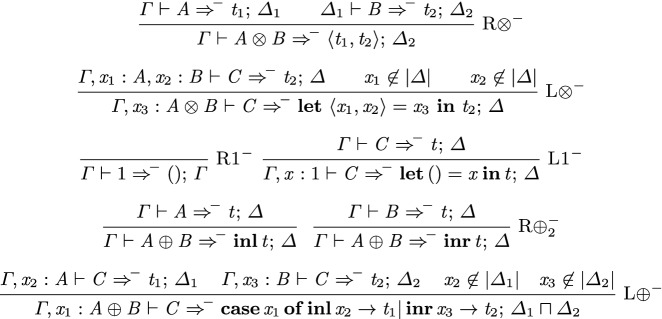



The $\textsc {L}\oplus ^{-}$ rule synthesises the left and right branches of a case statement that may use resources differently. The output context therefore takes the *greatest lower bound* ($\sqcap$) of $\varDelta _{{\mathrm {1}}}$ and $\varDelta _{{\mathrm {2}}}$. We elide definition of context $\sqcap$ as it has the same shape as $\sqcup$ for contexts (Definition 2), just replacing $\sqcup$ with $\sqcap$ on grades.

As an example of $\sqcap$, consider the semiring of intervals over natural numbers and two judgements that could be used as premises for the (L$\oplus ^{-}$) rule:
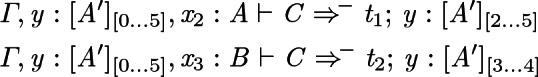


where $t_1$ uses *y* such that there are 2–5 uses remaining and $t_2$ uses *y* such that there are 3–4 uses left. To synthesise  the output context must be pessimistic about what resources are left, thus we take the greatest-lower bound yielding the interval $[ 2\dots 4 ]$ here: we know *y* can be used at least twice and at most 4 times in the rest of the synthesised program.

This completes subtractive synthesis. We conclude with a key result, that synthesised terms are well-typed at the type from which they were synthesised:

#### Lemma 2

**(Subtractive synthesis soundness).** For all $\varGamma$ and $A$ then:i.e. $t$ has type $A$ under context $\varGamma - \varDelta$, that contains just those linear and graded variables with grades reflecting their use in $t$. The appendix [22] provides the proof.

### Additive Resource Management

We now propose a dual *additive* resource management approach. Additive synthesis also uses the input-output context approach, but where output contexts describe exactly which assumptions were used to synthesise a term, rather than which assumptions are still available. Additive synthesis rules are read bottom-up, with $\varGamma \vdash A \Rightarrow ^{\!+} t ;\, \varDelta$ meaning that from the type $A$ we synthesise a term $t$ using exactly the assumptions $\varDelta$ that originate from the input context $\varGamma$.

We unpack the rules, starting with variables:For a linear assumption, the output context contains just the variable that was synthesised. For a graded assumption $x : [ A ]_{ r }$, the output context contains the assumption graded by 1. To synthesise a variable from a graded assumption, we must check that the use is compatible with the grade. The subtractive approach handled this rule (GrVar$^{-}$) by a constraint $\exists s .\, r \sqsupseteq s + 1$. Here however, the point at which we check that a graded assumption has been used according to the grade takes place in the $\textsc {L}\square ^{+}$ rule, where graded assumptions are bound:Here, $t$ is synthesised under a fresh graded assumption $x_{{\mathrm {2}}} : [ A ]_{ r }$. This produces an output context containing $x_{{\mathrm {2}}}$ with some grade *s* that describes how $x_{{\mathrm {2}}}$ is used in $t$. An additional premise requires that the original grade *r* approximates either *s* if $x_{{\mathrm {2}}}$ appears in $\varDelta$ or 0 if it does not, ensuring that $x_{{\mathrm {2}}}$ has been used correctly. For the $\mathbb {N}$-semiring with equality as the ordering, this would ensure that a variable has been used exactly the number of times specified by the grade.

Right and left rules for $\multimap$ have a similar shape to the subtractive calculus:

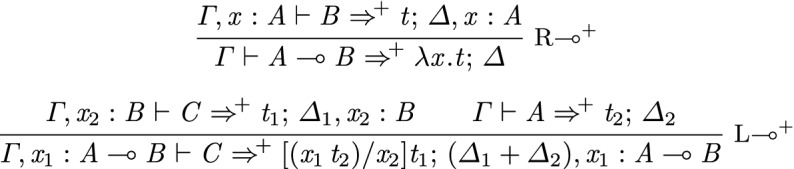



Synthesising an abstraction (R$\multimap ^{+}$) requires that $x : A$ is in the output context of the premise, ensuring that linearity is preserved. Likewise for application (L$\multimap ^{+}$), the output context of the first premise must contain the linearly bound $x_{{\mathrm {2}}} : B$ and the final output context must contain the assumption being used in the application $x_{{\mathrm {1}}} : A \multimap B$. This output context computes the *context addition* (Definition 1) of both output contexts of the premises $\varDelta _{{\mathrm {1}}} + \varDelta _{{\mathrm {2}}}$. If $\varDelta _{{\mathrm {1}}}$ describes how assumptions were used in $t_{{\mathrm {1}}}$ and $\varDelta _{{\mathrm {2}}}$ respectively for $t_{{\mathrm {2}}}$, then the addition of these two contexts describes the usage of assumptions for the entire subprogram. Recall, context addition ensures that a linear assumption may not appear in both $\varDelta _{{\mathrm {1}}}$ and $\varDelta _{{\mathrm {2}}}$, preventing us from synthesising terms that violate linearity.

As in the subtractive calculus, we avoid duplicating left rules to match graded assumptions by giving a synthesising version of dereliction:The fresh linear assumption $y : A$ must appear in the output context of the premise, ensuring it is used. The final context therefore adds to $\varDelta$ an assumption of $x$ graded by 1, accounting for this use of $x$ (temporarily renamed to *y*).

Synthesis of a promotion is considerably simpler in the additive approach. In subtractive resource management it was necessary to calculate how resources were used in the synthesis of $t$ before then applying the scalar context multiplication by the grade *r* and subtracting this from the original input $\varGamma$. In additive resource management, however, we can simply apply the multiplication directly to the output context $\varDelta$ to obtain how our assumptions are used in $[ t ]$:As in the subtractive approach, the right and left rules for products, units, and sums follow fairly straightforwardly from the resource scheme:

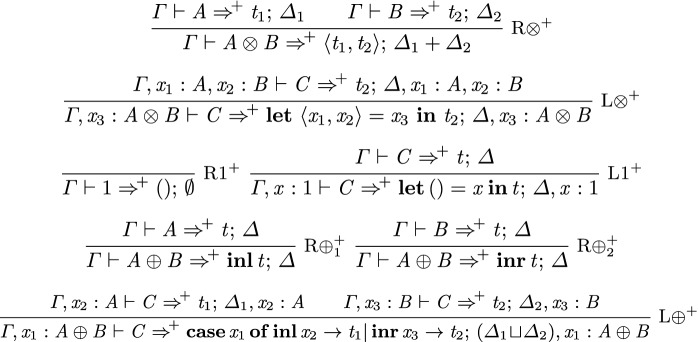



Rule (L$\oplus ^{+}$) takes the least-upper bound of the premise’s output contexts (Definition 2).

#### Lemma 3

**(Additive synthesis soundness).** For all $\varGamma$ and $A$:The appendix [22] provides the proof.

**Additive Pruning.** As seen above, the additive approach delays checking whether a variable is used according to its linearity/grade until it is bound. We hypothesise that this can lead additive synthesis to explore many ultimately ill-typed (or *ill-resourced*) paths for too long. Subsequently, we define a “pruning” variant of any additive rules with multiple sequenced premises. For (R$\otimes ^{+}$) this is:

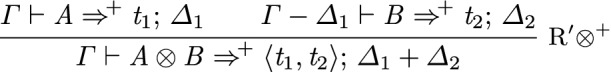



Instead of passing $\varGamma$ to both premises, $\varGamma$ is the input only for the first premise. This premise outputs context $\varDelta _{{\mathrm {1}}}$ that is subtracted from $\varGamma$ to give the input context of the second premise. This provides an opportunity to terminate the current branch of synthesis early if $\varGamma - \varDelta _{{\mathrm {1}}}$ does not contain the necessary resources to attempt the second premise. The (L$\multimap ^{+}$) rule is similarly adjusted.

#### Lemma 4

**(Additive pruning synthesis soundness).** For all $\varGamma$ and $A$:The appendix [22] provides the proof.

### Focusing

The two calculi provide a foundation for a synthesis algorithm. However, in their current forms, both synthesis calculi are highly non-deterministic: for each rule there are multiple rules which may be applied to synthesise the premise(s).

We apply the idea of *focusing* [3] to derive two *focusing calculi* which are equivalent to the former in expressivity, but with a reduced degree of non-determinism in the rules that may be applied. Focusing is a proof search technique based on the idea that some rules are *invertible*, i.e. whenever the premises of a rule are derivable, the conclusion is also derivable. Rules with this property can be applied eagerly in the synthesis of a term. When we arrive at a goal whose applicable rules are not invertible, we *focus* on either the goal type or a particular assumption by applying a chain of non-invertible rules until we reach a goal to which invertible rules can be applied. The appendix [22] gives focusing versions of the two calculi, which form the basis of our implementation. The proofs for the soundness of these focusing calculi can also be found in the appendix.

## Evaluation

Prior to evaluation, we made the following hypotheses about the relative performance of the additive versus subtractive approaches:

1. Additive synthesis should make fewer calls to the solver, with lower complexity theorems (fewer quantifiers). Dually, subtractive synthesis makes more calls to the solver with higher complexity theorems (more quantifiers);
2. For complex problems, additive synthesis will explore more paths as it cannot tell whether a variable is not well-resourced until closing a binder; additive pruning and subtractive will explore fewer paths as they can fail sooner.
3. A corollary of the above two: simple examples will likely be faster in additive mode, but more complex examples will be faster in subtractive mode.

**Methodology.** We implemented our approach as a synthesis tool for Granule, integrated with its core tool. Granule features ML-style polymorphism (rank-0 quantification) but we do not address polymorphism here. Instead, programs are synthesised from type schemes treating universal type variables as logical atoms. We discuss additional details of the implementation at the end of this section.

To evaluate our synthesis tool we developed a suite of benchmarks comprising Granule type schemes for a variety of operations using linear and graded modal types. We divide our benchmarks into several classes of problem:

- **Hilbert**: the Hilbert-style axioms of intuitionistic logic (including SKI combinators), with appropriate $\mathbb {N}$ and $\mathbb {N}$-interval grades where needed (see, e.g., *S* combinator in Example 1 or coproduct elimination in Example 2).
- **Comp**: various translations of function composition into linear logic: multiplicative, call-by-value and call-by-name using ! [17], I/O using ! [28], and coKleisli composition over $\mathbb {N}$ and arbitrary semirings: e.g. $\forall r, s \in \mathcal {R}$:
  $$\begin{aligned} \textit{comp-}{} \textit{coK}_{\mathcal {R}} : \square _{ r } ( \square _{ s } A \multimap B ) \multimap ( \square _{ r } B \multimap C ) \multimap \square _{ r * s } A \multimap C \end{aligned}$$
- **Dist**: distributive laws of various graded modalities over functions, sums, and products [23], e.g., $\forall r \in \mathbb {N}$, or $\forall r \in \mathcal {R}$ in any semiring, or $r = [ 0 ... \infty ]$:
  $$\begin{aligned} \textit{pull}_\oplus : ( \square _{ r } A \oplus \square _{ r } B ) \multimap \square _{ r } ( A \oplus B ) \quad \;\;\; \textit{push}_\multimap : \square _{ r } ( A \multimap B ) \multimap \square _{ r } A \multimap \square _{ r } B \end{aligned}$$
- **Vec**: map operations on vectors of fixed size encoded as products, e.g.:
  $$\begin{aligned} \!\! \textit{vmap}_5 : \square _{ 5 } ( A \multimap B ) \multimap ( ( ( ( A \otimes A ) \otimes A ) \otimes A ) \otimes A ) \multimap ( ( ( ( B \otimes B ) \otimes B ) \otimes B ) \otimes B ) \end{aligned}$$
- **Misc**: includes Example 3 (information-flow security) and functions which must share or split resources between graded modalities, e.g.:
  $$\begin{aligned} \!\! \textit{share}: \square _{ 4 } A \multimap \square _{ 6 } A \multimap \square _{ 2 } ( ( ( ( ( A \otimes A ) \otimes A ) \otimes A ) \otimes A ) \multimap B ) \multimap ( B \otimes B ) \end{aligned}$$

The appendix [22] lists the type schemes for these synthesis problems (32 in total). We found that Z3 is highly variable in its solving time, so timing measurements are computed as the mean of 20 trials. We used Z3 version 4.8.8 on a Linux laptop with an Intel i7-8665u @ 4.8 Ghz and 16 Gb of RAM.

**Table 1.**
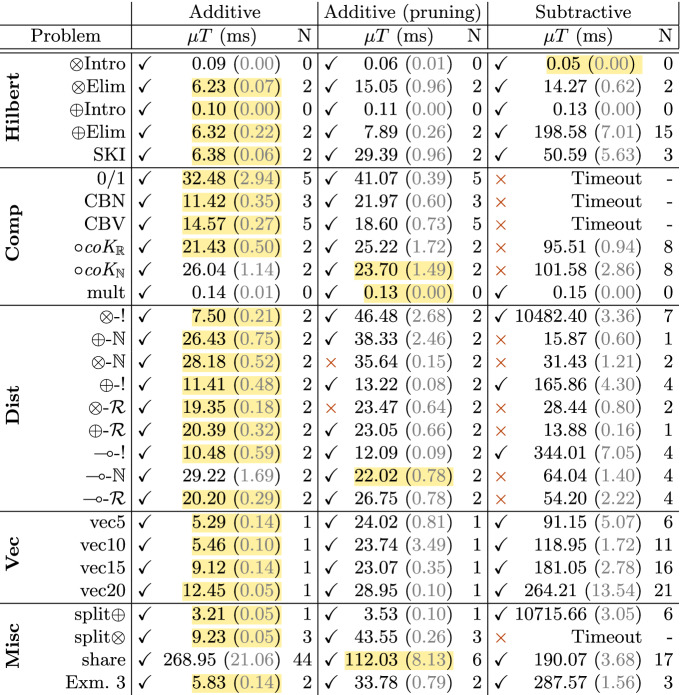
Results. $\mu {T}$ in *ms* to 2 d.p. with standard sample error in brackets

**Results and Analysis.** For each synthesis problem, we recorded whether synthesis was successful or not (denoted $\checkmark$ or  ), the mean total synthesis time ($\mu {T}$), the mean total time spent by the SMT solver ($\mu \textsc {smt}$), and the number of calls made to the SMT solver (N). Table 1 summarises the results with the fastest case for each benchmark highlighted. For all benchmarks that used the SMT solver, the solver accounted for 91.73%–99.98% of synthesis time, so we report only the mean total synthesis time $\mu {T}$. We set a timeout of 120 s.

*Additive vs. Subtractive.* As expected, the additive approach generally synthesises programs faster than the subtractive. Our first hypothesis (that the additive approach in general makes fewer calls to the SMT solver) holds for almost all benchmarks, with the subtractive approach often far exceeding the number made by the additive. This is explained by the difference in graded variable synthesis between approaches. In the additive, a constant grade 1 is given for graded assumptions in the output context, whereas in the subtractive, a fresh grade variable is created with a constraint on its usage which is checked immediately. As the total synthesis time is almost entirely spent in the SMT solver (more than 90%), solving constraints is by far the most costly part of synthesis leading to the additive approach synthesising most examples in a shorter amount of time.

Graded variable synthesis in the subtractive case also results in several examples failing to synthesise. In some cases, e.g., the first three *comp* benchmarks, the subtractive approach times-out as synthesis diverges with constraints growing in size due to the maximality condition and absorbing behaviour of $[ 0 ... \infty ]$ interval. In the case of $\textit{coK-}\mathcal {R}$ and $\textit{coK-}\mathbb {N}$, the generated constraints have the form $\forall r. \exists s. r \sqsupseteq s + 1$ which is not valid $\forall r \in \mathbb {N}$ (e.g., when $r = 0$), which suggests that the subtractive approach does not work well for polymorphic grades. As further work, we are considering an alternate rule for synthesising promotion with constraints of the form $\exists s . s = s' * r$, i.e., a multiplicative inverse constraint.

In more complex examples we see evidence to support our second hypothesis. The *share* problem requires a lot of graded variable synthesis which is problematic for the additive approach, for the reasons described in the second hypothesis. In contrast, the subtractive approach performs better, with $\mu {T} = 190.07$ ms as opposed to additive’s 268.95 ms. However, additive pruning outperforms both.

*Additive Pruning.* The pruning variant of additive synthesis (where subtraction takes place in the premises of multiplicative rules) had mixed results compared to the default. In simpler examples, the overhead of pruning (requiring SMT solving) outweighs the benefits obtained from reducing the space. However, in more complex examples which involve synthesising many graded variables (e.g. *share*), pruning is especially powerful, performing better than the subtractive approach. However, additive pruning failed to synthesis two examples which are polymorphic in their grade ($\otimes$-$\mathbb {N}$) and in the semiring/graded-modality ($\otimes$-$\mathcal {R}$).

Overall, the additive approach outperforms the subtractive and is successful at synthesising more examples, including ones polymorphic in grades and even the semiring itself. Given that the literature on linear logic theorem proving is typically subtractive, this is an interesting result. Going forward, a mixed approach between additive and additive pruning may be possible, selecting the algorithm, or even the rules, depending on the class of problem. Exploring this, and further optimisations and improvements, is further work.

**Additional Implementation Details.** Constraints on resource usage are handled via Granule’s existing symbolic engine, which compiles constraints on grades (for various semirings) to the SMT-lib format for Z3 [30]. We use the LogicT monad for backtracking search [26] and the Scrap Your Reprinter library for splicing synthesised code into syntactic “holes”, preserving the rest of the program text [10]. The implementation of the rule for additive dereliction ($\textsc {der}^{+}$) requires some care. A naïve implementation of this rule would allow the construction of an infinite chain of dereliction applications, by repeatedly applying the rule to the same graded assumption, as the correct usage of the assumption’s grade is only verified after it has been used to synthesise a sub-term. Our solution is to simply disallow immediate consecutive applications of the dereliction rule in additive synthesis, requiring that another rule be applied between multiple applications of the dereliction rule to any assumption. If no other rules can be applied, then the branch of synthesis is terminated.

## Discussion

**Further Related Work.** Before Hodas and Miller [20], the problem of resource non-determinism was first identified by Harland and Pym [19]. Their solution delays splitting of contexts at a multiplicative connective. They later explored the implementation details of this approach, proposing a solution where proof search is formulated in terms of constraints on propositions. The logic programming language Lygon [1] implements this approach.

Our approach to synthesis implements a *backward* style of proof search: starting from the goal, recursively search for solutions to subgoals. In contrast to this, *forward* reasoning approaches attempt to reach the goal by building subgoals from previously proved subgoals until the overall goal is proved. Pfenning and Chaudhuri consider forward approaches to proof search in linear logic using the *inverse method* [11] where the issue of resource non-determinism that is typical to backward approaches is absent [8, 9].

Non-idempotent intersection types systems have a similar core structure resembling the linear $\lambda$-calculus with quantitative aspects akin to grading [6]. It therefore seems likely that the approaches of this paper could be applied in this setting and used, for example, as way to enhance or even improve existing work on the inhabitation problem for non-idempotent intersection types [5]: a synthesised term gives a proof of inhabitation. This is left as further work, including formalising the connection between non-idempotent intersections and grading.

**Next Steps and Conclusions.** Our synthesis algorithms are now part of the Granule toolchain with IDE support, allowing programmers to insert a “hole” in a term and, after executing a keyboard shortcut, Granule tries to synthesise the type of the hole, pretty-printing generated code and inserting it at the cursor.

There are various extensions which we are actively pursuing, including synthesis for arbitrary user-defined indexed data types (GADTs), polymorphism, and synthesis of recursive functions. We plan to study various optimisations to the approaches considered here, as well as reducing the overhead of starting the SMT solver each time by instead running an “online” SMT solving procedure. We also plan to evaluate the approach on the extended linear logical benchmarks of Olarte et al. [31]. Although our goal is to create a practical program synthesis tool for common programming tasks rather than a general purpose proof search tool, the approach here also has applications to automated theorem proving.

## Footnotes

1 https://github.com/granule-project/granule/releases/tag/v0.8.0.0.
